# One-Seventh of Patients with COVID-19 Had Olfactory and Gustatory Abnormalities as Their Initial Symptoms: A Systematic Review and Meta-Analysis

**DOI:** 10.3390/life10090158

**Published:** 2020-08-22

**Authors:** Hsin Chi, Nan-Chang Chiu, Chun-Chih Peng, Chao-Hsu Lin, Yu-Lin Tai, Ming-Dar Lee, Yu-Jyun Cheng, Boon Fatt Tan, Chien-Yu Lin

**Affiliations:** 1 Department of Pediatrics, MacKay Children’s Hospital and MacKay Memorial Hospital, Taipei 10449, Taiwan; chi.4531@mmh.org.tw (H.C.); ncc88@mmh.org.tw (N.-C.C.); pengcc4566@gmail.com (C.-C.P.); 2 Department of Medicine, MacKay Medical College, New Taipei City 25160, Taiwan; 3 Department of Pediatrics, Hsinchu MacKay Memorial Hospital, Hsinchu 30071, Taiwan; 3099@mmh.org.tw (C.-H.L.); superlof@gmail.com (Y.-L.T.); 4554@mmh.org.tw (M.-D.L.); 4569@mmh.org.tw (Y.-J.C.); 4 Department of Biological Science and Technology, National Chiao-Tung University, Hsinchu city 30010, Taiwan; 5 Department of Pediatric Infectious Disease, National Taiwan University Hospital Hsinchu Branch, Hsinchu city 30059, Taiwan; wenfa80@gmail.com

**Keywords:** COVID-19, novel coronavirus, SARS-CoV-2, anosmia, ageusia

## Abstract

Coronavirus disease 2019 (COVID-19) patients exhibited protean clinical manifestations. Olfactory and gustatory abnormalities (anosmia and ageusia) were observed in COVID-19 patients, but the reported prevalence varied. In this systematic review, the prevalence of olfactory and gustatory abnormalities (OGA) was evaluated in laboratory-confirmed COVID-19 patients. On 8 May 2020, 14,506 articles were screened, while 12 of them were enrolled. A total of 1739 COVID-19 patients were analyzed, with a wide range of prevalence observed (5.6–94%). The pooled prevalence was 48.5% with high heterogeneity (*I*^2^, 98.8%; *p* < 0.0001). In total, 15.5% had OGA as their first symptom (*I*^2^, 22.6%; *p* = 0.27) among the patients analyzed. Contradictory to COVID-19 negative controls, patients with COVID-19 had a higher risk of OGA (odds ratio, 5.3; *I*^2^, 66.5%; *p* = 0.03). In conclusion, approximately half of COVID-19 patients had OGA, and one-seventh of them had OGA as their initial symptoms. OGA were cardinal symptoms of COVID-19, which may serve as clues for early diagnosis. Diagnostic testing for SARS-CoV-2 was suggested in patients with OGA during the COVID-19 pandemic to ensure timely diagnosis and appropriate quarantine.

## 1. Introduction

The emerging crisis of the coronavirus disease 2019 (COVID-19) was caused by Severe Acute Respiratory Syndrome Coronavirus 2 (SARS-CoV-2) and became a huge burden to the world. As of July 2020, there were more than 100 million infected people and 500 thousand deaths worldwide [1]. Approximately 5% of patients required invasive care and the case fatality rate may be 40% in these patients [2]. The optimal strategies to combat COVID-19 varied across countries, however, timely diagnosis, prompt quarantine, and treatment play crucial roles for successful management [3,4].

The incubation period was approximately five days, while fever and respiratory symptoms were major symptoms of the disease [5,6]. However, the full disease entity remained largely unclear. Olfactory and gustatory abnormalities (anosmia and ageusia), which were not reported in earlier studies in China, were observed in March 2020. These symptoms were easily overlooked, but a wide range of prevalence was reported. In Mao’s study, approximately 5% of COVID-19 patients had OGA, while two-thirds had OGA in Luers’ study [7,8]. The prevalence of OGA among patients with COVID-19 caught our attention, thus, the possible role of OGA as red flags to initiate diagnostic testing needed to be further clarified. Therefore, this systematic review was conducted to investigate the prevalence of OGA in COVID-19 patients.

## 2. Materials and Methods

### 2.1. Study Design and Literature Search

This study was approved by the Institutional Review Board of the MacKay Memorial Hospital, Taipei, Taiwan (approval number, 20MMHIS140e) and was conducted in accordance with the Preferred Reporting Items for Systematic Reviews and Meta-analyses (PRISMA) guidelines [9,10]. Comprehensive keywords were used, such as “COVID-19”, “COVID-2019”, “severe acute respiratory syndrome coronavirus 2”, “2019-nCoV”, “2019nCoV”, “SARS-CoV-2”, and “Wuhan” with Boolean operators and MeSH terms. Electronic medical databases were searched from inception to 8 May 2020, including PubMed/Medline, EMBASE, Cumulative Index to Nursing and Allied Health Literature, National Digital Library of Theses and Dissertations in Taiwan database, Art Image Indexing Service on the Internet Database (Chinese database), and the Cochrane database. The search was independently performed by two authors, and disagreements were resolved through a discussion with the third author. No constraints were placed on language, year of publication, and participant characteristics to ensure a comprehensive search and identify the maximum number of potential articles. Authors were contacted to obtain additional information when necessary.

### 2.2. Study Selection and Data Extraction

Studies investigating “olfactory”, “gustatory”, “smell”, “taste”, “anosmia”, ”hyposmia”, “ageusia”, and “hypogeusia” were analyzed. The exclusion criteria were as follows: duplicate publications, irrelevant articles, studies where the infection status was not clearly confirmed, studies that did not evaluate clinical outcomes, simple case reports, and review articles. Primary outcomes were the prevalence of OGA, respectively.

Furthermore, two authors independently appraised the selected articles and extracted the following data: name of the first author, study country, participant population, demographic data, OGA prevalence, OGA tests, onset time, recovery time, and COVID-19 test results. In case of disagreement between the two authors, consensus was reached through a discussion with the third author. Quality assessments were conducted independently by two authors based on the domains of selection, ascertainment, causality, and reporting.

### 2.3. Statistical Analyses

If meta-analysis was performed, a random-effect regression model was utilized assuming that the true effect size was not the same. Heterogeneity was further quantified using Cochran’s Q test and *I*^2^ statistics. A *p* value less than 0.05 was considered statistically significant. MedCalc (MedCalc software, Belgium) v18 was used for statistical analyses.

## 3. Results

**Figure 1:**
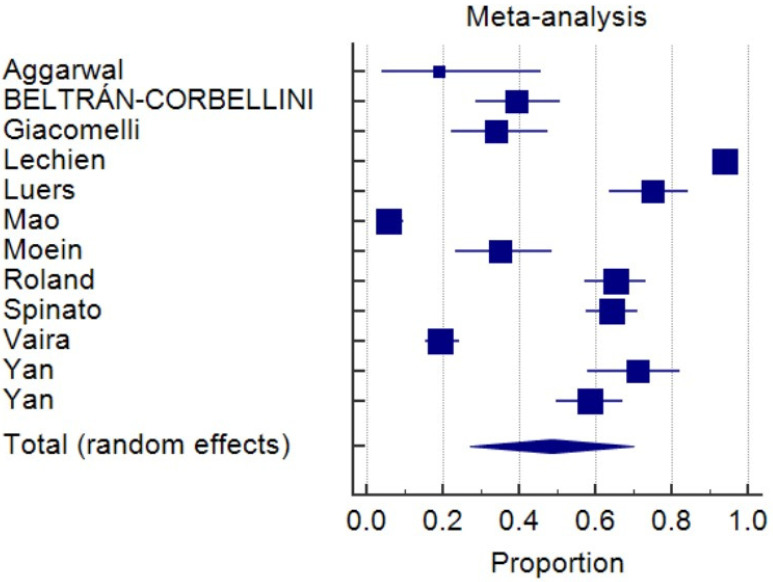
Forest plot of prevalence of olfactory and gustatory abnormalities. The pooled prevalence was 48.5% (*I*^2^, 98.8%; *p* < 0.0001).

**Figure 2:**
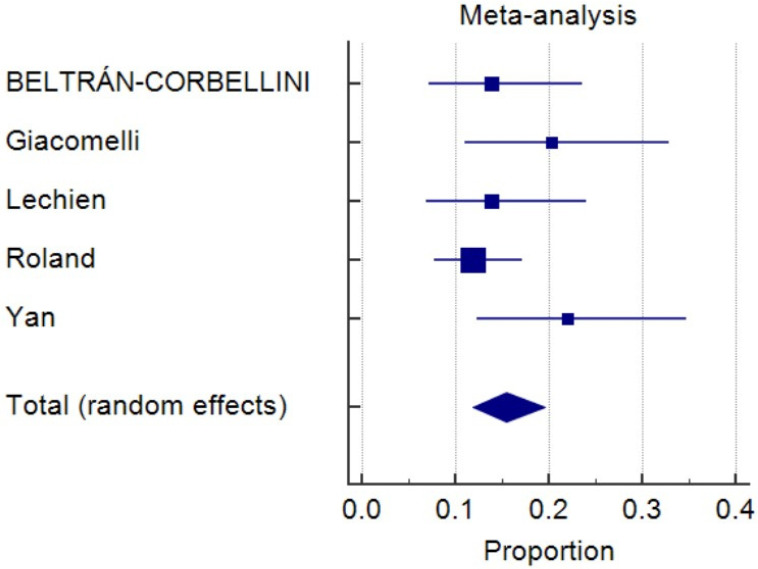
Forest plot of prevalence of olfactory and gustatory abnormalities as initial symptoms (pooled prevalence, 15.5%; *I*^2^, 22.6%; *p* = 0.27).

**Figure 3:**
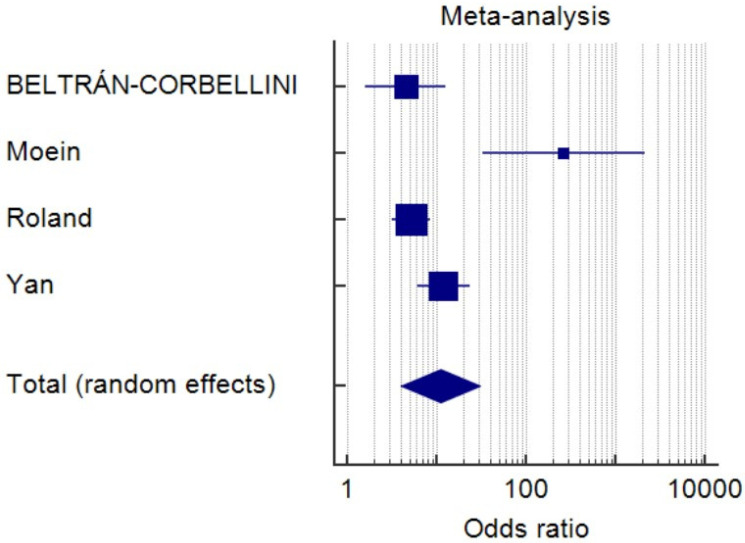
Forest plot of enrolled studies investigating the odds ratio of olfactory and gustatory abnormalities in COVID-19 patients and controls without COVID-19. (Odds ratio, 5.3; *I*^2^, 66.5%; *p* = 0.03).

As of 8 May 2020, 14,506 non-duplicated articles were selected from the medical research database, and the titles and abstracts of all articles were screened. Twelve studies fulfilling the inclusion and exclusion criteria were included in the final systematic review (Table 1) [7,8,11,12,13,14,15,16,17,18,19,20]. Six studies were conducted in Europe, four in the USA, one in China, and one in Iran. A total of 1739 participants were identified, and the pooled prevalence was 48.5% (*I*^2^, 98.8%; *p* < 0.0001) (Figure 1). Five studies involving 471 individuals reported the onset of OGA, and 15.5% of the patients had OGA as initial symptoms (Figure 2) (pooled prevalence, 15.5%; *I*^2^, 22.6%; *p* = 0.27). Four studies comparing COVID-19 patients and negative controls possessed a higher risk of OGA (Figure 3) (odds ratio, 5.3; *I*^2^, 66.5%; *p* = 0.0297). The funnel plots were plotted and showed no significant asymmetry (Supplementary Figures S1–S3).

## 4. Discussion

Based on our systematic review, there was a high prevalence of OGA in COVID-19 patients, and 15.5% had OGA as their first symptom. Compared with controls, patients with COVID-19 had a higher risk of OGA (odds ratio, 5.3). OGA were cardinal symptoms of COVID-19, which might serve as a clue for early diagnosis. Patients with OGA should receive diagnostic testing for SARS-CoV-2 during the COVID-19 pandemic to initiate early diagnosis, appropriate quarantine, and treatment.

COVID-19 patients had protean clinical manifestations. Moreover, several diseases may cause OGA. OGA were observed in patients with allergic rhinitis and respiratory virus infection, such as influenza and other coronaviruses [12]. We found a higher risk of OGA in patients with COVID-19 and OGA was the initial presentation in one-seventh of patients. Therefore, patients exhibiting OGA should undergo SARS-CoV-2 testing during the COVID-19 pandemic. However, the exact pathogenesis of COVID-19 remained largely unclear. OGA may result from direct virus invasion of the central nervous system or peripheral nerves [21]. Angiotensin-converting enzyme 2 (ACE2) was believed to be involved in the pathogenesis of COVID-19. Increased expression of ACE2 receptors in olfactory-specific horizontal basal cells and sustentacular cells were reported; thus, retrograde injury of the peripheral nervous system may cause OGA in COVID-19 patients. Further studies are required to clarify the underpinning pathogenesis of OGA in COVID-19.

There were some systematic reviews investigating the prevalence of OGA in patients with COVID-19 published recently [22,23,24]. In total, 43.93–52.73% patients were reported to have OGA [22]. Half patients had OGA in Hoang’s study [23]. Costa et al. also had similar findings (56.4–60.7%) [24]. Two recent studies investigating the prevalence of OGA in Italy were reported by Vaira. They found the prevalence of OGA was 73.6% (53/72) and 74.2% (256/345) [25,26]. Our findings were consistent with these reports and the high prevalence of OGA in COVID-19 was reemphasized. Furthermore, we analyzed the proportion of OGA as the initial symptoms, which was not clearly reported previously, and found one-seventh (15.5%) of patients with COVID-19 had OGA as their first symptoms. The presence of OGA before onset of fever, cough, and other respiratory symptoms provided clues for early diagnosis of COVID-19. Timely diagnosis and prompt quarantine ensured adequate containment of disease and was helpful to identify possible infected people in areas with limited diagnostic resources.

The wide range of reported prevalence may be attributed to the diagnostic methods. Most OGA were self-reported and might be easily overlooked. Changes of nasal tactile sensitivity were reported in patients with OGA and may provide an objective and reliable diagnostic reference for physicians [27]. During the COVID-19 pandemic, home quarantine was an important strategy to contain disease and onsite tests for OGA in hospitals were not always available. Objective tests were a challenge in such situations. Vaira et al. developed a self-administered olfactory and gustatory test using solutions with different concentrations to determine the olfactory and gustatory thresholds at home [28]. There was no significant difference between this remote evaluation and hospital validation tests. This practice was feasible and more reliable. Furthermore, they found 256 COVID-19 patients (74.2%) had self-reported OGA in the multi-center study in Italy; they performed objective tests in patients without self-reported OGA. Twenty-seven patients (30.3%) were found to have objectively mild hyposmia [26]. These findings indicated the potential risk of underestimating of OGA in patients of COVID-19 and emphasized the important role of objective tests in future studies.

The high prevalence of OGA was observed in our systematic review, and high heterogeneity was found. An extraordinary low prevalence (5.6%) of OGA was reported in Mao’s study conducted in China. Further subgroup analysis by geographic areas was performed, which revealed a 55.8% prevalence in Europe (*I*^2^, 99.12%; *p* < 0.0001) and 54.9% in the USA (*I*^2^, 87.18%; *p* = 0.0004). Further studies are warranted to investigate possible differences between races and countries.

Compared with other respiratory viruses, SARS-CoV-2 infection had a longer disease course and the duration of OGA was investigated [26]. A long duration of olfactory abnormality was observed and approximately 70% patients had OGA after 1 month. However, the severity of olfactory abnormality improved rapidly after the first 10 days. Gustatory dysfunction was less durable and 55% patients returned to normal gustatory function after the third week. Interestingly, the severity of OGA was not correlated to the severity of COVID-19 illness; however, a longer duration of OGA > 7 days might have higher risk of severe COVID-19. Further studies are required to clarify the correlation and underpinning pathophysiology between OGA and COVID-19.

Our study was subject to some limitations. First, OGA were self-reported symptoms, and some studies were retrospective. Recalling bias may exist which calls for a randomized prospective study. Second, nasal and tongue mucosa biopsy and other biomarkers were not tested, and the underlying pathophysiology between OGA and COVID-19 was not explored. Although our systematic review reported a strong correlation between OGA and COVID-19, further studies are warranted to elucidate the causal pathogenesis.

## 5. Conclusions

In conclusion, this systematic review and meta-analysis identified 1739 laboratory-confirmed COVID-19 patients, and we found that approximately half of the patients had OGA. Approximately one-seventh of the patients had OGA as their initial presentation; thus, the presence of OGA may provide a clue for early diagnosis. Compared with controls, patients with COVID-19 had higher risk of OGA. Diagnostic testing for SARS-CoV-2 was suggested in patients with OGA during the COVID-19 pandemic.

## Figures and Tables

**Table 1:** Demographic characteristics of enrolled studies investigating OGA.

Study [Ref.]	Country	Study Population	Study Participants, N	Patients with OGA, N (%)	OGA as Initial Presentation, N (%)	Controls, N	Controls with OGA, N (%)
Aggarwal [11]	USA	hospitalized patients	16	3 (18.8)			
Beltrán-Corbellini [12]	Spain	hospitalized patients	79	31 (39.2)	11 (13.9)	40	5 (12.5)
Giacomelli [13]	Italy	hospitalized patients	59	20 (33.9)	12 (20.3)		
Lechien [14]	Belgium, France, Spain, Italy	mild-to-moderate patients	385	362 (94)			
Luers [7]	Germany	not reported	72	54 (75)	10 (13.9)		
Mao [8]	China	hospitalized patients	214	12 (5.6)			
Moein [15]	Iran	mild, moderate, severe	60	21 (35)		60	11 (18.3)
Roland [16]	USA	hospitalized and outpatients	145	95 (66)		157	42 (26.8)
Spinato [17]	Italy	home patients	202	130 (64.4)	24 (11.9)		
Vaira [18]	Italy	not reported	320	62 (19.4)			
Yan [19]	USA	hospitalized and outpatients	59	42 (71.2)	13 (22)	203	35 (17.2)
Yan [20]	USA	hospitalized and outpatients	128	75 (58.6)			
Total			1739	907 (48.5)	70 (15.5)	460	93 (20.2)

Abbreviations: OGA—olfactory and gustatory abnormalities; Controls—COVID-19 negative controls; Ref.—reference.

## Supplementary Materials

The following are available online at https://www.mdpi.com/2075-1729/10/9/158/s1, Figure S1: Funnel plot of enrolled studies investigating the prevalence of olfactory and gustatory abnormalities; Figure S2: Funnel plot of enrolled studies investigating the prevalence of olfactory and gustatory abnormalities as initial symptoms; Figure S3: Funnel plot of enrolled studies investigating the prevalence of olfactory and gustatory abnormalities between COVID-19 and controls.
