# Supplementary material for: One-Seventh of Patients with COVID-19 Had Olfactory and Gustatory Abnormalities as Their Initial Symptoms: A Systematic Review and Meta-Analysis
Source: Life (Basel). 2020 Aug 22;10(9):158. doi: 10.3390/life10090158 (PMC7554793; doi:10.3390/life10090158)
Supplement: Supplementary file 1 [file life-10-00158-s001.pdf]

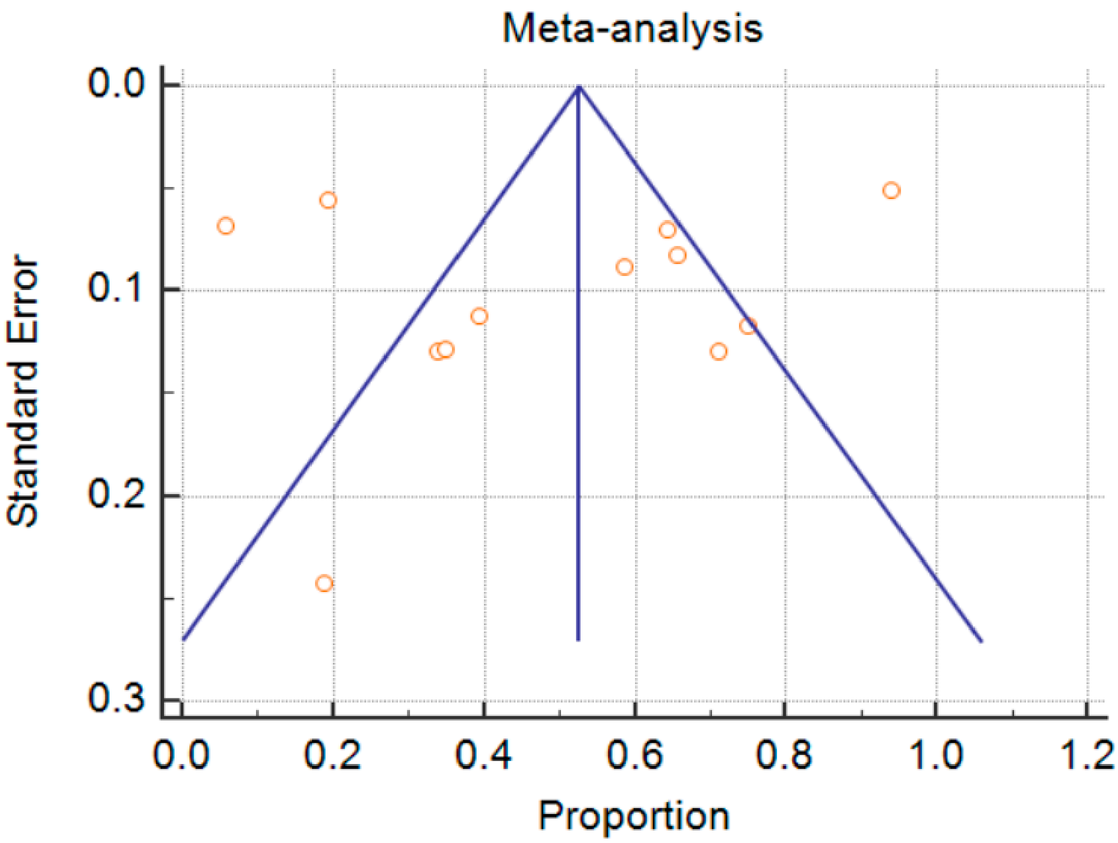

Supplementary Figure S1. funnel plot of enrolled studies investigating the prevalence of olfactory and gustatory abnormalities.

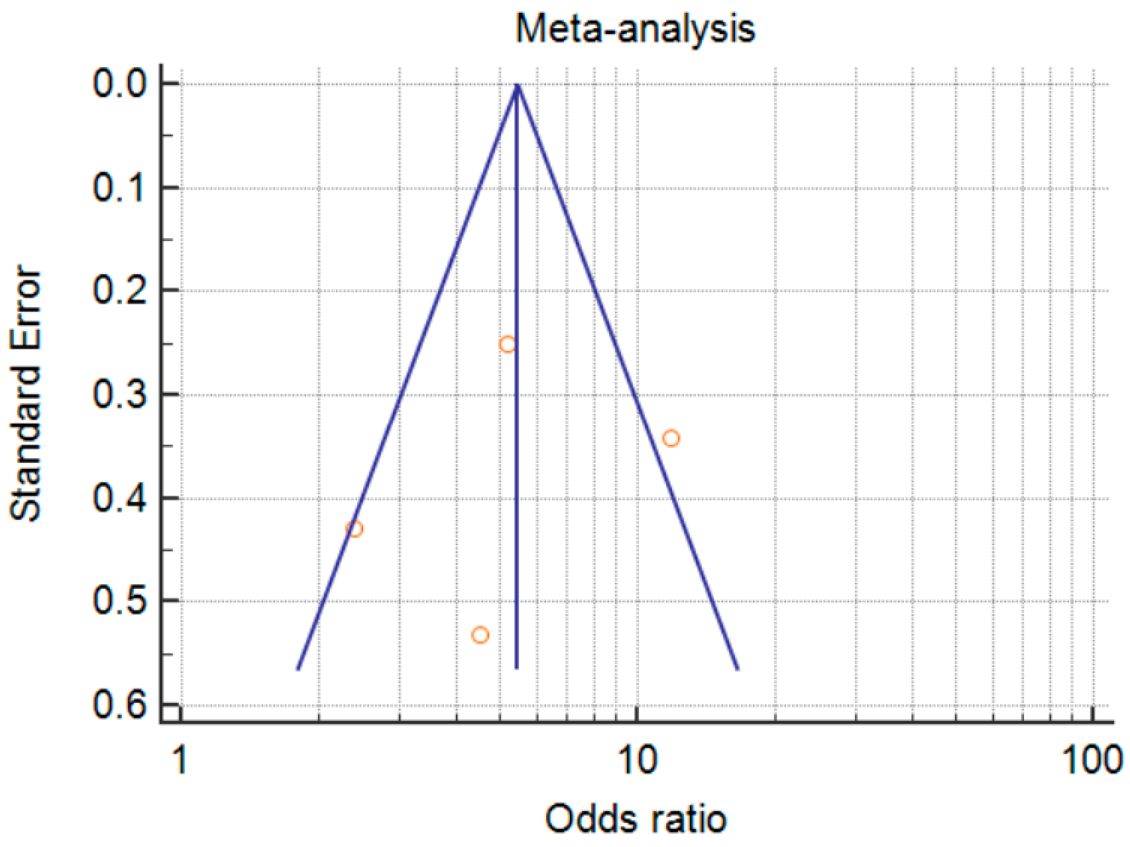

Supplementary Figure S2. funnel plot of enrolled studies investigating the prevalence of olfactory and gustatory abnormalities as initial symptoms.

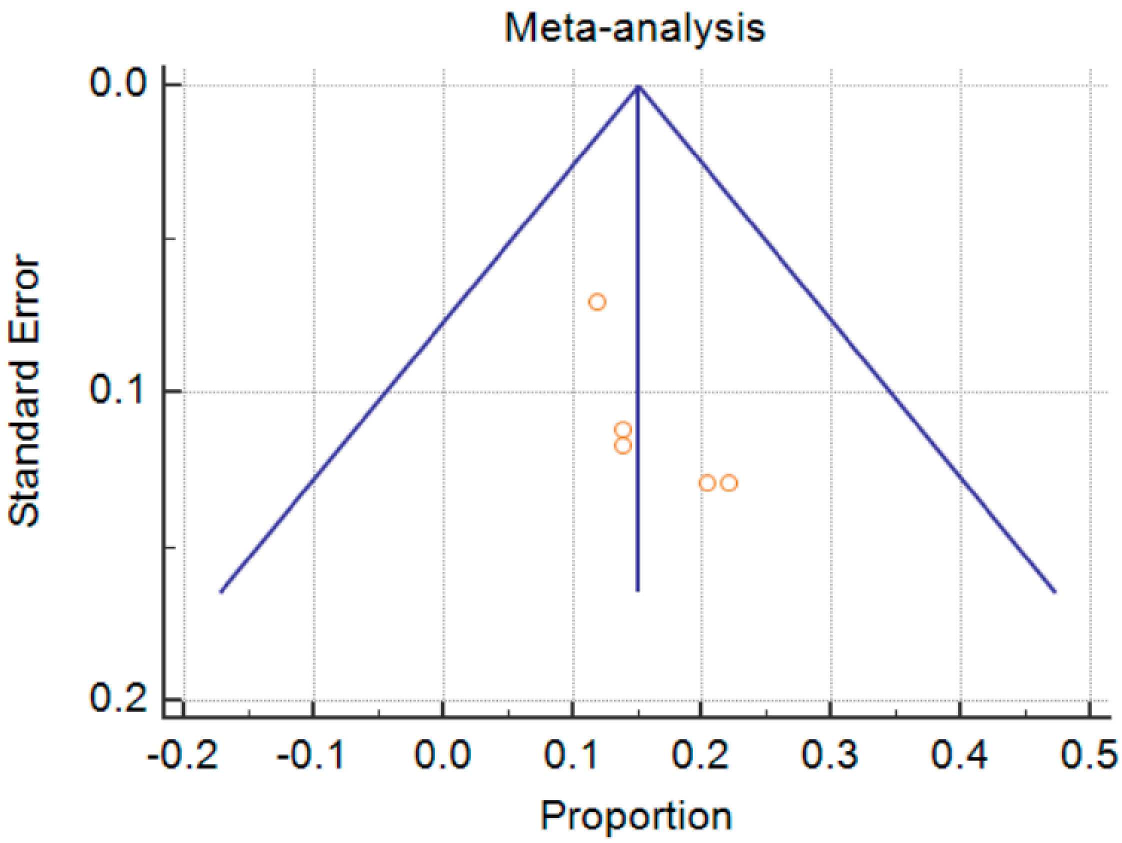

Supplementary Figure S3. funnel plot of enrolled studies investigating the prevalence of olfactory and gustatory abnormalities between COVID-19 and controls.
